# Amyloid-β Triggers the Release of Neuronal Hexokinase 1 from Mitochondria

**DOI:** 10.1371/journal.pone.0015230

**Published:** 2010-12-16

**Authors:** Leonardo M. Saraiva, Gisele S. Seixas da Silva, Antonio Galina, Wagner S. da-Silva, William L. Klein, Sérgio T. Ferreira, Fernanda G. De Felice

**Affiliations:** 1 Programa de Bioquímica e Biofísica Celular, Instituto de Bioquímica Médica, Universidade Federal do Rio de Janeiro, Rio de Janeiro, Rio de Janeiro, Brazil; 2 Department of Neurobiology and Physiology, Northwestern University, Evanston, Illinois, United States of America; Mental Health Research Institute of Victoria, Australia

## Abstract

Brain accumulation of the amyloid-β peptide (Aβ) and oxidative stress underlie neuronal dysfunction and memory loss in Alzheimer's disease (AD). Hexokinase (HK), a key glycolytic enzyme, plays important pro-survival roles, reducing mitochondrial reactive oxygen species (ROS) generation and preventing apoptosis in neurons and other cell types. Brain isozyme HKI is mainly associated with mitochondria and HK release from mitochondria causes a significant decrease in enzyme activity and triggers oxidative damage. We here investigated the relationship between Aβ-induced oxidative stress and HK activity. We found that Aβ triggered HKI detachment from mitochondria decreasing HKI activity in cortical neurons. Aβ oligomers further impair energy metabolism by decreasing neuronal ATP levels. Aβ-induced HKI cellular redistribution was accompanied by excessive ROS generation and neuronal death. 2-deoxyglucose blocked Aβ-induced oxidative stress and neuronal death. Results suggest that Aβ-induced cellular redistribution and inactivation of neuronal HKI play important roles in oxidative stress and neurodegeneration in AD.

## Introduction

Hexokinase (HK) catalyzes the first step of glycolysis, i.e., the ATP-dependent phosphorylation of glucose to glucose-6P (G6P), with concomitant generation of ADP. Although HK is generally known as a key glycolytic enzyme, in neurons and other cell types HK activity also regulates vital cellular processes, including ATP synthesis and apoptosis [1]–[3].

In the brain, HKI is the major isozyme present [4], being mainly (∼70–90%) associated with the outer mitochondrial membrane. Release of HK from mitochondria is known to cause a severe decrease in enzyme activity [5], [6]. Interestingly, mitochondrial-bound hexokinase I (m-HKI) activity in neurons has been shown to be neuroprotective, maintaining adequate glutathione levels, inducing neurite outgrowth, and preventing neuronal oxidative damage [6]–[8]. The activity and specific subcellular localization of neuronal m-HKI is of great significance to protect neurons from different insults.

We have previously demonstrated that neuronal m-HKI reduces hyperglycemia-induced generation of excessive ROS through an ADP recycling mechanism [8]. In the mitochondrial membrane, HKI is bound to the voltage-dependent anion channel (VDAC), which is associated with the ADP/ATP carrier. HKI benefits from preferential access to ATP produced in the mitochondria, while local ADP generation by HKI facilitates the exchange of ADP and ATP through the inner mitochondrial membrane [8], [9]. This enhances mitochondrial oxidative phosphorylation and reduces monoelectronic oxygen reduction that gives rise to excessive ROS generation. Thus, m-HK1 may play an important antioxidant role in the brain.

Several cellular features of the brain suggest that it is highly sensitive to oxidative stress [10]. Abnormally elevated ROS levels have been implicated in the age-related impairment of long-term potentiation (LTP), a well-known model for synaptic plasticity and learning [11]. It is known that excessive ROS levels are implicated in the molecular etiology of Alzheimer's disease (AD) [12]–[14]. Elevated ROS levels can be selectively dysfunctional in AD, a disease characterized by memory loss.

Previous investigations have shown that different aggregated forms of the amyloid-β peptide (Aβ) stimulate ROS production in neurons [14], [15]. Accumulation of Aβ in AD brains is thought to underlie neuronal dysfunction and memory loss, being centrally implicated in AD pathogenesis [16]. In particular, soluble protein oligomers are currently thought to be emerging toxins in Alzheimer's [17]–[19] and other amyloid diseases [18], [20].

We now report that Aβ triggers neuronal oxidative stress by interfering with m-HKI activity and subcellular localization. Exposure of mature cortical neurons to Aβ caused a decrease in m-HKI activity and its detachment from mitochondria. Aβ oligomers further induced mitochondrial dysfunction and caused a marked reduction in neuronal ATP levels, indicating an impairment of energy metabolism. By causing a cellular redistribution of HKI, Aβ instigates an abnormal increase in mitochondrial ROS generation that is prevented by 2-deoxyglucose (2-DOG). Results establish a novel cellular mechanism underlying oxidative stress and neurodegeneration in AD.

## Methods

### Materials

Aβ_1-42_ and Aβ_1-40_ were purchased from Bachem (Torrance, CA). ADP, ATP, horseradish peroxidase, carbonyl cyanide *p-*trifluoromethoxyphenylhydrazone (FCCP), 2-deoxyglucose (2-DOG), *Leuconostoc Mesenteroid*'s glucose-6 phosphate dehydrogenase (G6PDH), Percoll, β-NAD+, bovine serum albumin and poly-L-lysine were from Sigma-Aldrich (St. Louis, MO). Neurobasal medium, B27 supplement, Live/Dead cell viability assay kit and CM-H_2_DCFDA were from Invitrogen Molecular Probes (Eugene, OR). Goat polyclonal anti-cytochrome c oxidase and monoclonal anti-hexokinase-1 antibodies were from Santa Cruz Biotechnology (Santa Cruz, CA). Anti-cyclophilin B rabbit polyclonal antibody was from Affinity Bio Reagents (Golden, CO). SuperSignal West Femto Maximum Sensitivity substrate was from Pierce (Rockford, IL). All other reagents were of the highest analytical grade available.

### Aβ preparation

Aβ_1-42_ was freshly dissolved from the lyophilized powder in a 50% (v/v) solution of trifluoroethanol (TFE) in phosphate-buffered saline (PBS), as described [21]–[23]. Amyloid aggregation was triggered by dilution of small aliquots from this stock solution into PBS (resulting in ≤ 0.5% residual TFE) to yield final concentrations of 5 µM or 20 µM Aβ.

Alternatively, Aβ oligomers were prepared as previously described [14], [24], [25]. Briefly, the peptide was dissolved to 1 mM in hexafluoro-2-propanol and stored in aliquots as a dried film at −80°C after solvent evaporation. The film was resuspended in DMSO to a final concentration of 5 mM. The solution was diluted to 100 µM in ice-cold PBS and left at 4°C overnight. The solution was then centrifuged at 14,000×g for 10 min at 4°C and the supernatant was collected, transferred to a clean tube and stored at 4°C for a maximum of 24 hours until use. Aβ_1-40_ monomer solution was prepared in aliquots as a dried hexafluoro-2-propanol film and stored at -80°C. The peptide film was dissolved in undiluted, sterile DMSO to make a 5 mM solution. The solution was diluted to 100 µM with Ham's F-12 medium without glutamine and immediately centrifuged (14,000×g/10 min/4°C) to remove insoluble aggregates. The supernatant was transferred to a clean tube and used immediately.

### Brain mitochondrial isolation, determination of HK activity and isolation of neuronal cytosolic and mitochondria fractions

Mitochondria were isolated from forebrains by conventional differential centrifugation as previously described [8]. m-HKI activity was determined using an enzymatic assay coupled to *Leuconostoc Mesenteroid*'s G6PDH activity, monitoring hexokinase activity indirectly by NADH formation at λ = 340 nm [8]. Cytosolic and mitochondria-enriched fractions from primary cultures of rat cortical neurons were isolated as described [8].

### Neuronal cultures and reactive oxygen species (ROS) formation

Cortices from 14-day-old rat embryos were dissected and cultured as previously described [22], [26] with minor modifications. Cells were plated on glass coverslips previously coated with 1.5 µg/ml poly-L-lysine in Neurobasal medium supplemented with B27 and cultures were maintained at 37°C in a humidified 5% CO_2_ atmosphere for 14–18 days prior to use. ROS formation was evaluated in live cortical neurons using 2 µM CM-H_2_DCFDA [14]. Probe fluorescence was analyzed using NIH Image J software [27] as described [14]. 16 images were analyzed in each experimental condition (carried out in triplicate wells in each of three independent experiments using different neuronal cultures) and were combined to allow quantitative estimates of changes in ROS levels.

### Neuronal viability and MTT assays

Aβ_1-42_ (20 µM), in the absence or in the presence of 30 mM 2-DOG, was added to cultures and was kept in the medium for 48 hours. Control cultures consisting of neurons cultured in growth medium alone or in the presence of residual TFE (0.5% v/v) were also prepared. Cell viabilities in cultures were assessed using the Live/Dead kit. Live neurons were identified by green calcein fluorescence and dead neurons were identified by red propidium iodide fluorescence. Percentages of live neurons are expressed relative to the total number of neurons in each field. Five randomly chosen fields independent fields were imaged on a Nikon Eclipse TE300 microscope and analyzed in different experimental conditions (carried out in at least triplicate wells in each of three independent experiments using different cultures).

MTT (3-[4,5-dimethyl-thiazol-2-yl]-2,5-diphenyl tetrazolium bromide) assay (Boehringer Mannheim, Indianapolis, IN) was used to evaluate the cellular metabolic redox activity. Nineteen-day old hippocampal neuronal cultures were used. Three independent experiments (each with 6 wells per experimental condition) were carried out with different neuronal cultures.

### Measurement of intracellular ATP levels

ATP contents in 19-day old rat cortical neurons were analyzed by ion-paired reverse phase liquid chromatography. Cultures were exposed to 500 nM ADDLs or vehicle for 12 hours. Medium was then removed and cells were washed twice with cold PBS. Liquid nitrogen was used to disrupt cells and stop cellular metabolism. The plates were kept in an ice bath until the liquid nitrogen evaporated completely. Cells were then homogenized with 6% trichloroacetic acid, neutralized by adding a small aliquot of a 1M Tris solution, and centrifuged at 20,800×g for 5 min at 4°C. Protein contents were determined using the BCA method. Aliquots were injected into an HPLC system using a Supelguard column (Supelco) coupled to a Supercosil C-18 carrier (particle size of 5 µm, Supelco) column. Isocratic elution was performed at a flow-rate of 1 mL/min at room temperature with 50 mM KH_2_PO^4^ buffer, pH 6.0/methanol (90/10) and 4 mM tetrabutyl ammonium bromide. ATP levels were measured by UV absorbance at 254 nm. ATP peaks were identified by co-injecting 2 nmols of ATP in an independent run. Results were expressed by normalizing peak areas by the total amount of protein obtained.

### Determination of intracellular glucose-6P content

Aβ_1-42_ (5 µM), in the the presence of 30 mM 2-DOG, was added to cultures after 7 days in vitro and was kept in the medium for 24 hours. Cells were scraped in 200 µL of 6% (v/v) trichloroacetic acid per plate (35 mm2 dishes). The extract was neutralized by adding 80 µL of 1.0 M Tris solution. G6P levels were determined using an enzymatic assay coupled to G6PDH activity [28], [29]. Intracellular G6P levels were expressed as nmols/mg protein.

### Western blot analysis

Aliquots from mitochondria-enriched and cytosolic cellular fractions were normalized for protein content (25 µg protein/lane) and separated on 12% SDS-PAGE gels followed by blotting onto nitrocellulose membranes. Hexokinase levels were determined from total, unfractionated neuronal extracts separated on 12% SDS-PAGE gels and blotted onto nitrocellulose membranes. Membranes were incubated with anti-HK1 (1∶500 dilution), anti-cytochrome c oxidase (1∶500 dilution, used as a loading control for mitochondria-enriched fractions) and anti-cyclophilin B (1∶4,000 dilution, used as a loading control for cytosolic fractions) antibodies. Protein bands were visualized by staining with horseradish peroxidase-labeled secondary antibody (1∶100,000) followed by enhanced chemiluminescence detection.

## Results

### Aβ induces inhibition of neuronal mitochondrial-bound hexokinase I (m-HKI) by interference with subcellular localization

**Figure 1:**
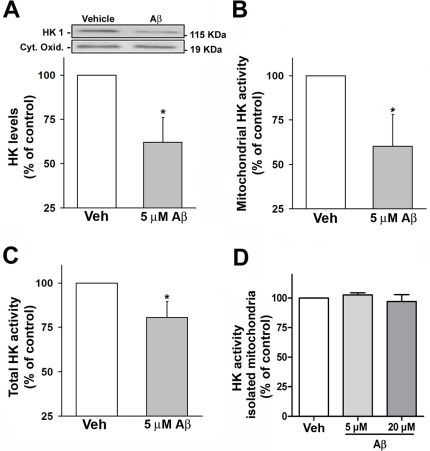
Aβ inhibits HKI by triggering the release of m-HKI from mitochondria. Panel A: Western immunoblot analysis of HKI levels in mitochondrial fractions from cultured cortical neurons exposed to 5 µM Aβ for 24 hours. Cytochrome c oxidase was used as a loading control. Bars correspond to means ± SE from at least three independent experiments carried out in duplicate. Panel B: HK activity as measured in mitochondrial enriched fractions from cultured cortical neurons exposed to 5 µM Aβ for 24 hours. Bars correspond to means ± S.D. from three independent experiments carried out in duplicate. Panel C: HK1 activity as measured in cortical neurons exposed to 5 µM Aβ for 24 hours. Bars represent the means ± SD from three independent experiments carried out in duplicate. (*) indicates a statistically significant (p<0.005) difference relative to control (vehicle-treated) cultures. Panel D: HK activity in mitochondria isolated from adult rat brains was measured in the absence or in the presence of Aβ (5 µM or 20 µM; 1 hour). Values represent means ± SD of the activity measured in two independent experiments carried out in triplicate.

Brain HKI is mainly (∼70–90%) associated with the mitochondrial membrane and release of HK from mitochondria causes a significant decrease in enzyme activity [5], [6]. To test whether Aβ interferes with the subcellular localization of neuronal m-HKI, we measured HKI protein levels in mitochondrial fractions by western immunobloting. HKI levels were reduced by ∼40% in the mitochondrial fraction from Aβ-treated neurons (Fig. 1A). In agreement with the decrease in levels of mitochondrial-bound hexokinase, the activity determined in the mitochondria-enriched fraction from Aβ-treated neurons was significantly (∼40%) lower than the activity measured in mitochondria from vehicle-treated neurons (Fig. 1B). We also found that 5 µM Aβ induced ∼ 20% decrease in total neuronal HKI activity (Fig. 1C). These results show that Aβ interferes with the subcellular localization of HK, causes the release of HKI from mitochondria. In order to investigate the effect of Aβ on m-HKI activity, we have next proceeded checking whether Aβ directly inhibits HK. m-HKI activity was then measured in mitochondria isolated from adult rat brains incubated with Aβ. Aβ did not directly inhibit hexokinase, even at a high concentration (20 µM) (Fig. 1D). This suggests that Aβ only has an impact on HKI when the enzyme is associated with the mitochondrial membrane in the neuronal cellular context.

### Oligomers of the Aβ peptide decrease mitochondrial-bound HKI and impair neuronal energy metabolism

**Figure 2:**
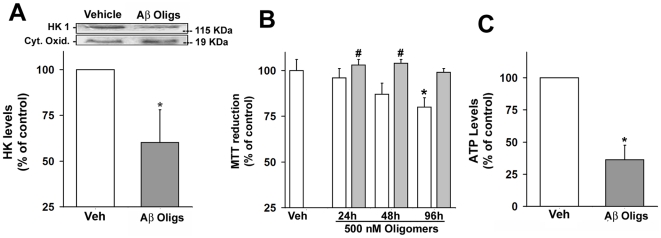
Effects of Aβ oligomers on neuronal m-HKI and ATP levels. Panel A: Aβ oligomers triggers the release of m-HKI from mitochondria. Western immunoblot analysis of HKI levels in mitochondrial fractions from cultured cortical neurons exposed to 500 nM Aβ oligomers (ADDLs) (D) for 24 hours. Cytochrome c oxidase was used as a loading control. Bars correspond to means ± SE from at least three independent experiments carried out in duplicate. Panel B: Time-dependent toxicity of ADDLs (white bars) or Aβ monomers (gray bars) to mature hippocampal neurons measured using the MTT reduction assay. Values represent the means ± SD of the relative absorbance measured using 6 wells for each experimental condition for three independent experiments. (*) indicates a statistically significant (p<0.005) difference relative to control cultures. Panel C: Aβ oligomers decrease ATP levels in mature cortical neurons. Cultures were exposed to vehicle or 500 nM ADDLs for 12 hours. Values represent means ± SD of the relative levels measured in five independent experiments.

Because soluble Aβ oligomers (also known as ADDLs) have recently been recognized as the proximal neurotoxins in AD [17]–[19], we next investigated whether a defined preparation of soluble Aβ oligomers would instigate HKI release from mitochondria. Indeed, HKI levels were 40% reduced in the mitochondrial fraction from neurons treated with 500 nM ADDLs for 24 hours (Fig. 2A). Results demonstrate that Aβ oligomers instigate neuronal HKI release from mitochondria.

The impact of Aβ oligomers on mitochondrial redox activity was further evaluated using the MTT reduction assay in our model of mature hippocampal neuronal cultures. Neurons were exposed to ADDLs or monomers for 24, 48 or 96 h with ADDLs (Fig. 2B). After 24 h, no significant toxicity was observed in the presence of ADDLs or monomers, used as a control. Only at longer incubation times (48 and 96 h), ADDLs caused a progressive decrease in MTT reduction, while Aβ monomers had no effect (Fig. 2B).

ATP is essential for the excitability and survival of neurons, as well as for protein phosphorylation reactions that mediate synaptic signaling and associated changes in neuronal structure and function [28]. Because the results presented above have indicated mitochondrial impairment in ADDL-treated neurons, we next measured ATP levels in cortical neurons treated with 500 nM ADDLs for 12 hours, well before an effect on mitochondrial redox activity could be observed (Fig. 2B). Results show a marked decrease in ATP levels in ADDL-treated neurons (Fig. 2C), substantiating the neuronal energy metabolism impairment caused by Aβ oligomers.

### Hexokinase activity blocks Aβ-induced generation of reactive oxygen species (ROS)

**Figure 3:**
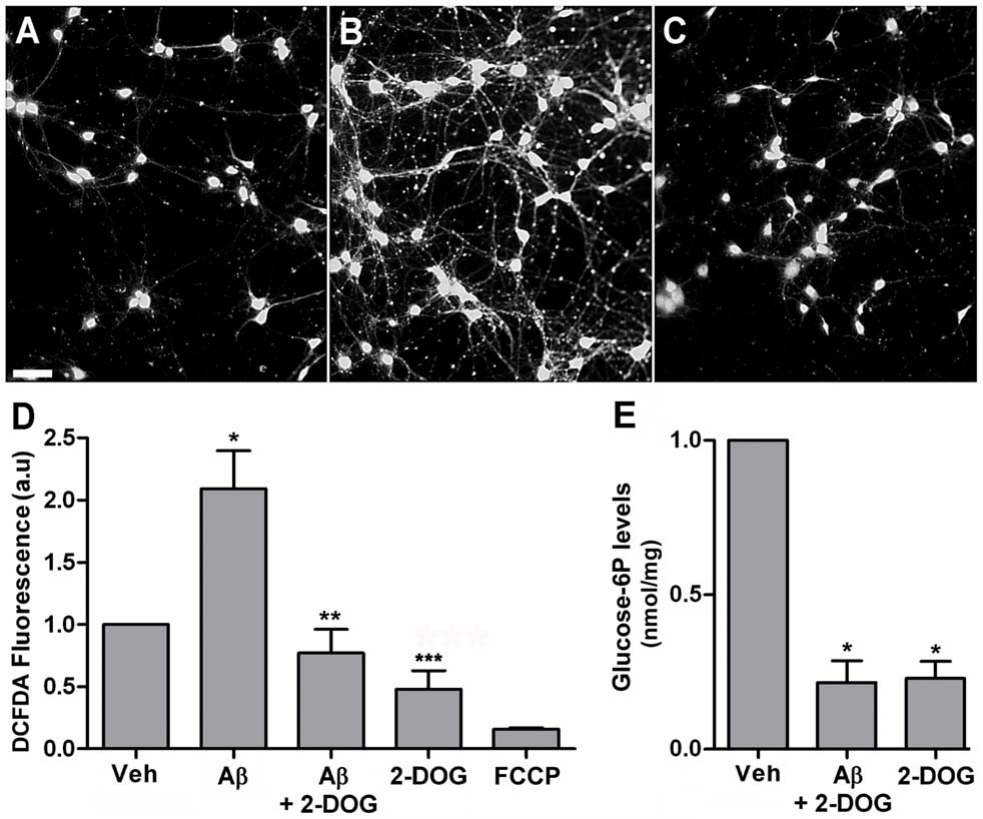
2-DOG blocks Aβ-induced neuronal ROS formation. Representative DCF fluorescence images from control (vehicle-treated) cultures (Panel A), cultures exposed to 5 µM Aβ for 6 hours (Panel B) or cultures exposed to 5 µM Aβ+30 mM 2-DOG for 6 hours (Panel C). When present, 2-DOG was added 10 minutes before Aβ. In order to allow direct comparison between ROS levels, identical conditions and exposure times for image acquisition were employed for all experimental conditions. Panel D: Quantitative analysis of DCF fluorescence from five independent experiments (∼ 1,500 cells analyzed per experimental condition in each experiment) using ImageJ software (NIH Windows version). (*) indicates a statistically significant (p<0.005) difference relative to control cultures. (**) and (***) indicates a statistically significant (p<0.005) difference relative to Aβ-treated cultures. Scale bar corresponds to 100 µm. Panel E: 2-DOG decreases neuronal G6P levels in neurons exposed to Aβ. Glucose-6P levels were measured in control cortical neurons or neurons exposed to 5 µM Aβ+30 mM 2-DOG or 30 mM 2-DOG. Values represent means ± SD from three experiments (carried out in duplicate) using independent neuronal cultures. (*) indicates a statistically significant (p<0.001) difference relative to control cultures.

We have previously shown that impaired m-HKI activity results in increased ROS levels in neurons [8]. In order to investigate the possible role of m-HKI in neuronal oxidative stress induced by Aβ, intracellular ROS levels were evaluated in cortical neurons treated with 5 µM Aβ or vehicle for 6 hours, in the absence or presence of 2-deoxyglucose (2DOG), an alternative substrate of hexokinase. Phosphorylation of 2-DOG by hexokinase gives rise to the formation of 2-deoxyglucose 6-phosphate (2-DOG6P), which does not inhibit hexokinase activity in the same range as G6P and consequently preserves HK1 activity [4], [8], [29]. In agreement with previous findings [14], Aβ induced a significant increase in neuronal ROS levels (Fig. 3B, D). Interestingly, addition of 30 mM 2-DOG to the culture medium completely blocked the excessive intracellular ROS production induced by Aβ (Fig. 3C, D). Such results reveal an important antioxidant role of HKI in the prevention of Aβ-induced oxidative stress.

When neurons were treated with 5 µM FCCP (an uncoupler of oxidative phosphorylation that reduces mitochondrial membrane potential) in the presence or absence of Aβ, ROS levels became lower than those observed in control cultures (Fig. 3D), indicating that ROS formation in our experiments originates in mitochondria. We next measured intracellular G6P levels in cortical neurons exposed to Aβ, as G6P is a potent inhibitor of m-HKI at physiological concentrations and causes its release from brain mitochondria [29]. We found that treatment with 2-DOG, both in the absence and in the presence of Aβ greatly decreased neuronal G6P levels (Fig. 3E). Results indicate that the protective effects of 2-DOG may involve a decrease in intracellular G6P levels, thus counteracting the inhibition of HKI by Aβ and leading to decreased mitochondrial ROS production and death via enhanced ADP recycling.

### DOG blocks Aβ-induced neuronal death

**Figure 4:**
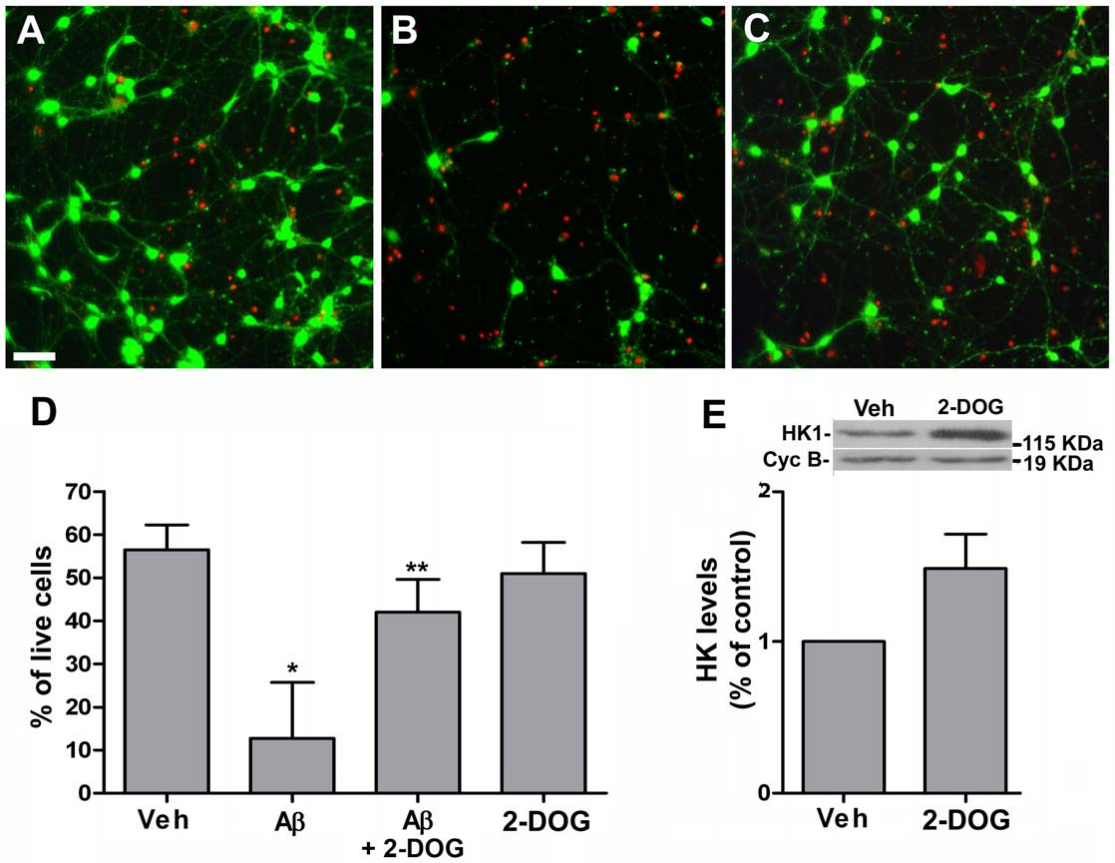
2-DOG blocks Aβ-induced neurodegeneration. Neuronal cortical cultures were maintained in the absence or in the presence of Aβ for 48 hours and cell viability was determined using the Live/Dead assay as described in “Materials and Methods”. Live cells are identified by green calcein fluorescence and dead cells are identified by red ethidium bromide fluorescence. Representative images from control (vehicle-treated) cultures (Panel A), cultures exposed to 20 µM Aβ (Panel B) or cultures treated with 20 µM Aβ+30 mM 2-DOG (Panel C). Panel D: Quantitative analysis of neuronal viability was carried out using Image J (described in “Methods”). A total of 3,000–4,000 cells were analyzed in each experimental condition. Bars show means ± SD from four different experiments (carried out in triplicate). (*) and (**) indicates a statistically significant (p<0.005 or p<0.01, respectively) difference relative to control cultures. Scale bar corresponds to 100 µm. Panel E: Effects of 2-DOG on neuronal HKI levels. Western immunoblot analysis of total HKI levels from cultured cortical neurons exposed to 30 mM 2-DOG for 48 hours. Cyclophilin B was used as a loading control. Bars correspond to means ± SD from four independent experiments.

Finally, we investigated whether 2-DOG protects neurons against Aβ-induced neurodegeneration. Cortical neurons were treated for 48 hours with Aβ in the absence or presence of 2-DOG and neuronal viability was determined using the Live/Dead assay. A marked neurodegeneration was observed in cultures exposed to 20 µM Aβ_1-42_ for 48 hours (Fig. 4). Remarkably, 2-DOG led to significant blockade of Aβ-induced neuronal death (Fig. 4). 2-DOG by itself had no effect on the viability of neurons in culture during 48 hours (Fig. 4D). Finally, we investigated if 2-DOG could interfere with HK1 levels. Increased total levels of HKI were found in cortical neurons treated with 30 mM 2-DOG (Fig. 4E), suggesting that the neuroprotective effect afforded by 2-DOG may also involve up-regulation of HKI levels.

## Discussion

In the present work we show that Aβ, the neurotoxin thought to be responsible for neuronal deterioration in Alzheimer's disease (AD), causes the release of hexokinase from mitochondria and a parallel inhibition of enzyme activity. Mitochondrial-bound hexokinase (m-HKI) regulates important cellular processes in neurons, including ATP synthesis, maintenance of glutathione levels and neurite outgrowth [6], [7], [30]. Importantly, we have previously shown that m-HKI participates in a local ADP recycling mechanism that is directly involved in the regulation of mitochondrial oxidative homeostasis, preventing neurons from oxidative damage [8]. Oxidative stress is an important facet of AD [12]–[14]. We have now established that the impact of Aβ on neuronal hexokinase triggers mitochondrial oxidative stress and neuronal death. Importantly, the glucose analogue, 2-DOG, prevented Aβ-induced inactivation of m-HKI, and blocked excessive mitochondrial ROS formation and neuronal death. Aβ-induced cellular redistribution and inactivation of m-HKI may play central roles in oxidative stress and neurodegeneration in AD.

While monomeric Aβ is not neurotoxic, the peptide exhibits a marked toxic gain-of-function upon self-association. Fibrillar forms of Aβ found in amyloid plaques are toxic to neurons [21], [31] and were until recently considered being mainly responsible for neuronal damage in AD. More recently, however, new evidence has emerged demonstrating that Aβ oligomers, aggregates that are soluble and much smaller than fibrils, are potent neurotoxins in AD pathogenesis [17], [18], causing neuronal dysfunction at much lower concentrations than fibrillar aggregates. Aβ oligomers accumulate in the brains of AD patients [32], [33] and have been linked to major AD pathology hallmarks, including neuronal tau hyperphosphorylation [24], oxidative stress [14], [15] and synapse deterioration [25]. Interestingly, oligomers from a non-disease related protein have been shown to mimic Aβ-induced tau hyperphosphorylation and neurodegeneration [26]. Aβ oligomers further inhibit long-term potentiation (LTP) [34], [35]. Our results reveal a novel deleterious neuronal impact of Aβ oligomers, causing m-HKI release from mitochondria (Fig. 2).

We also found that Aβ oligomers cause a marked reduction in neuronal ATP levels (Fig. 2). Impairment of brain energy metabolism is another major facet in AD that has been revealed by *in vivo* studies of glucose utilization [12], [36]. Brain energy metabolism is also altered in transgenic mouse models of AD that present Aβ deposition and elevated levels of Aβ oligomers [37], [38]. The brain relies almost exclusively on glucose as its source of energy, using approximately 25% of circulating sugar. Normal glucose levels have been shown to protect brain cells from apoptotic events [39], demonstrating that a fine regulation of metabolism is crucial to cellular survival under stress conditions. Glial cells are believed to take up a significant fraction of glucose from the blood and provide neurons with lactate and glucose-derived energy substrates to sustain their activity [40], [41]. Nonetheless, neurons also rely directly on glucose provided via the extracellular space by the cerebral circulation. Conversion of glucose to G6P and oxidative phosphorylation take place both in neurons and glial cells and generate the ATP required to drive many cellular processes, including the excitability and survival of neurons and intracellular signaling pathways related to neuronal plasticity [28]. Our finding that Aβ oligomers decrease neuronal ATP levels indicates that they are linked to the impairment of energy metabolism in neurons. This might, at least in part, explain the deficits in glucose utilization in AD brains, a condition believed to result in synaptic dysfunction, neuronal degeneration and cognitive impairment.

Interestingly, Aβ did not inhibit hexokinase activity measured in an isolated mitochondria preparation (Fig. 1D). Rather, inhibition of m-HKI was observed when cortical neurons in culture were treated with Aβ (Fig. 1B). This suggests that Aβ-induced inactivation of m-HKI only occurs when the enzyme is associated with the mitochondrial membrane in the neuronal cellular context. Further studies will be required to investigate whether Aβ directly interacts with m-HKI in neurons. An elegant study demonstrated that Aβ interacts with alcohol dehydrogenase in the mitochondria of AD patients and transgenic mice [42]. In addition, nicastrin, presenilin, APH-1 and PEN-2 form active gamma-secretase complexes in mitochondria [43], suggesting that Aβ can be physiologically formed in mitochondria.

Another possibility is that Aβ interaction with specific membrane receptors causes deregulation of intracellular signaling cascades that result in m-HKI inhibition. We have shown that Aβ oligomers induce abnormal Ca^2+^ influx and neuronal oxidative stress through aberrant activation of N-methyl-D-aspartate (NMDA) receptors [14]. Several lines of evidence suggest that Aβ interferes with Wnt/β-catenin signaling [44], [45], causing GSK-3β activation, a kinase abnormally active in AD and involved in tau phosphorylation [46], [24]. GSK-3β phosphorylates VDAC, causing HKII release from mitochondria in HeLa cells [47]. Akt activation is required to maintain hexokinase attachment to mitochondria [30]. Growth factors known to induce Akt activation, such as insulin and insulin growth factor, may increase HK association to VDAC, protecting against Aβ. Insulin in fact has recently been shown to protect synapses against Aβ oligomers [25].

Neuronal oxidative stress is a hallmark of AD pathology. Neurons in culture exposed to Aβ present increased markers of oxidative stress [14], [48], [49]. It is well established that mitochondrial ROS formation triggers early events in apoptosis, including mitochondrial swelling, cytochrome c release and caspase activation [12]. During aging, neurons are subject to increased oxidative stress and impaired energy metabolism, leading to dysfunction of proteins responsible for maintaining proper membrane excitability and subcellular Ca^2+^ dynamics [28]. Oxidative stress destabilizes Ca^2+^ homeostasis and renders neurons vulnerable to excitotoxicity and apoptosis [50]. Mitochondria preserve neuronal Ca^2+^ homeostasis by generating the ATP required to pump Ca^2+^ out of the cytoplasm and by buffering cytosolic Ca^2+^ loads needed during normal synaptic activity [51]. Aβ-induced mitochondrial dysfunction and oxidative stress (Fig. 3) may thus be of central importance to disrupt neuronal Ca^2+^ homeostasis.

Excessive ROS formation due to hyperpolarization of the mitochondrial membrane has also been shown to result from hyperglycemic neuronal injury, a condition in which m-HKI is inhibited by accumulation of G6P [8], [29], [52]. It is possible, therefore, that the decrease in intraneuronal G6P levels in the presence of 2-DOG (Fig. 3E) relieves the inhibition of m-HKI, sustaining a steady-state ADP recycling that sets down the mitochondrial membrane potential to lower levels and, consequently, protects neurons from Aβ-induced oxidative stress. Our finding that 2-DOG completely blocked Aβ-induced ROS formation and neuronal death (Figs. 3 and 4) further indicates that preserving neuronal m-HKI activity by preventing its release from mitochondria plays a key role in neuronal survival. Blocking the impact of Aβ on m-HKI may provide a novel therapeutic approach to prevent oxidative stress and neurodegeneration in AD.
